# Pressure-controlled ventilation-volume guaranteed mode improves bronchial mucus transport velocity in patients during laparoscopic surgery for gynecological oncology: a randomized controlled study

**DOI:** 10.1186/s12871-023-02343-2

**Published:** 2023-11-20

**Authors:** Chao Deng, Tao Xu, Xue-kai Wang, Deng-feng Gu

**Affiliations:** 1 Department of Anesthesiology, First Affiliated Hospital, Shihezi University, Shihezi, China; 2 Department of Rehabilitation, Tongji Hospital, Tongji Medical College, Huazhong University of Science and Technology, Wuhan, China

**Keywords:** Airway mucosa, Pressure-controlled ventilation-volume guaranteed, Volume-controlled ventilation, Bronchial mucus transport velocity, Laparoscopic surgery, Gynecological oncology

## Abstract

**Background:**

Mechanical ventilation during general anesthesia may impair airway mucosal function. This study aimed to investigate the effect of pressure-controlled ventilation-volume guaranteed (PCV-VG) on bronchial mucus transport velocity (BTV) in patients during laparoscopic surgery for gynecological oncology compared with volume controlled ventilation (VCV).

**Methods:**

66 patients undergoing elective a laparoscopic surgery for gynecological oncology. The patients were randomized into two group receiving either PCV-VG or VCV. a drop of methylene blue was placed on the surface of the airway mucosa under the bronchoscopeand, then the distance the dye movement was measured after 2, 4, and 6 min. Outcomes were assessed at T0 (5 min after endotracheal intubation and before initiation of pneumoperitoneum), T1 and T2 (1 and 2 h after stabilization of pneumoperitoneum respectively). BTV at T0, T1 and T2 was the primary outcome. Secondary outcomes included heart rate (HR), mean arterial pressure (MAP), body temperature, end**-**tidal CO2 pressure (PETCO2), tidal volume(V_T_), peak inspiratory pressure (PIP), mean inspiratory pressure (Pmean), respiratory rate (RR), and dynamic compliance (C_Dyn_) at T0, T1, and T2.

**Results:**

64 patients were included in the analysis. The median [interquartile range] BTV was significantly lower in VCV group at T1 and T2 that at T0 (*P* < 0.05). Furthermore, BTV was slightly reduced in PCV-VG compared with VCV. BTV in PCV-VG was significantly decreased at T2 compared with BTV at T0 (*P* < 0.05) and slightly decreased at T1 compared with T0(P > 0.05). Compared with the PCV-VG group, BTV in VCV group significantly decreased at T2 (*P* < 0.05). No participants experienced respiratory complications.

**Conclusions:**

PCV-VG is more suitable for patients undergoing laparoscopic surgery for gynecological oncology than VCV since it can protect mucociliary clearance function.

**Trial registration:**

This trial is registered on https://www.chictr.org.cn/ in Chinese Clinical Trial Registry (ChiCTR.2200064564: Date of registration 11/10/2022).

## Background

Airway mucosa is the most important natural defense system in the body and is considered the first line of defense of the respiratory system [1]. Airway mucosa can clear harmful particles and pathogenic microorganisms in the respiratory system [2]. Therefore, impaired function of airway mucosa can decrease mucociliary clearance ability, leading to respiratory infection, atelectasis, and other complications [3, 4]. Furthermore, airway mucosa is the most important natural defense system retained by the body after endotracheal intubation, and also the most sensitive part [5, 6]. The impact force of shear flow on the respiratory tract, change of airway pressure, and other factors during laparoscopic surgery for gynecological oncology can reduce airway mucociliary clearing function, thereby increase the risk of pulmonary infection [7, 8]. Therefore, an appropriate ventilation mode is necessary to minimize the respiratory system injury during laparoscopic surgery.

Pressure-controlled ventilation-volume guaranteed (PCV-VG) mode utilizes a decelerating flow and constant pressure to achieve volume control and pressure control. It has thus been widely used in clinical practice [9, 10]. The ventilator parameters change automatically with each breath of the patient to provide the target tidal volume (V_T_) without increasing airway pressure, which decreases the risk of barotrauma and respiratory system damage caused by mechanical ventilation [11]. However, the effect of PCV-VG ventilation mode on bronchial mucociliary clearance is unknown.

To bridge this data gap, in this study, we used bronchial mucus transport velocity (BTV) to quantify mucociliary clearance [12, 13], and measured BTV by direct observation of tracer motion length per unit time with the help of fiberoptic bronchoscopy [12]. Furthermore, we sought to compare the impact of PCV-VG or volume-controlled ventilation (VCV) on BTV in laparoscopic gynecological oncology surgery. We hypothesized that the PCV-VG mode of ventilation would maintain better BTV during laparoscopic surgery for gynecological oncology.

## Methods

### Study design

This was a single-center, prospective, patient and observer-blinded, randomized- controlled trial. This study was reported following the CONSORT reporting guidelines [14]. The whole study protocol was approved (Reference No. KJ2022-144–01;) by the Research Ethics Board of The First Affiliated Hospital of Shihezi University School of Medicine, China, on 13, September 2022. This trial is registered on https://www.chictr.org.cn/ in Chinese Clinical Trial Registry (ChiCTR.2200064564: Date of registration 11/10/2022). All participants provided an informed written consent form. Written informed consent was obtained from all participants at least the day before the surgery.

### Study population

First, all patients scheduled to undergo elective laparoscopic gynecologic tumor resection at the First Affiliated Hospital of Medical College, Shihezi University, China, from 12 October 2022 to 13 May 2023, were recruited. Next, only patients aged 40–75 years and met the American Society of Anesthesiologists’ (ASA) physical status I-II were included in the study. Patients with a history of respiratory disease, surgery, smoking, or recent use of drugs that can affect BTV (adrenergic receptor antagonists, hormones, anticholinergic drugs, theophylline, and catecholamines) predicted airway difficulty, and atopy were excluded from the study.

### Anesthesia procedures

The patients were not administered preoperative medications. Electrocardiogram, pulse oximetry, non-invasive blood pressure, and Bispectral Index Sensor (BIS, Aspect Medical Systems, Newton, MA, USA) were used to monitor all patients-Dash 3000 monitor, GE Company, Madison, USA. Anesthesia was induced using a bolus containing 0.05 mg/kg midazolam, 1 mg/kg propofol, 0.3 mg/kg sufentanil, and 1.5 mg/kg rocuronium after insertion of a peripheral intravenous cannula induction (unified model). Anesthesia was maintained through intravenous infusion of 50–150 ug/(kg·min) propofol and 0.05–0.1 g/kg· min remifentanil to maintain BIS within 40–60 and the mean arterial pressure within 20% of the pre-induction value. The patients also received intravenous injection of 0.7 mg/kg vecuronium to maintain muscle relaxation during anesthetization. Tracheal intubation was performed after muscle release. The cuff was inflated with 10 mL of air. The pressure of the tracheal intubation CUF was maintained at 20–30 cmH_2_O. The patients were tracheally intubated using an endotracheal tube (7.5 mm; interdermal [ID]). The tracheal tube balloon was fixed at a shallow position (about 2 cm below the glottis), and then the temperature of the nasopharynx was monitored.

### Study intervention

The patients received ventilation using the same type of Anesthesia Machine (Datex-Ohmeda-Avance CS2, GE Company, Madison, USA) after induction of anesthesia and intubation. Anesthesia machine parameters were similar under the two modes (VT; 8 ml /kg, inspiratory/expiratory ratio (I/E); 1:2, positive end-expiratory pressure (PEEP); 0, inspiratory fresh gas flow rate; 2.0 L/min with an inspired oxygen fraction (Fio2) of 0.4 in an oxygen-air. and respiratory rate (RR); initially set at 12 breath/min). Subsequently, RR was adjusted to maintain an end-tidal CO2 pressure (ETCO2) of 35–40 mmHg. The peak inspiratory pressure (PIP) and pneumoperitoneum pressure were set at 40cmH_2_O and 12 ± 2 mmHg, respectively. The patients were kept in the lithotomy position with the head low and hip high at a fixed head position of 30° from the bed.

### Measurements

**Fig. 1:**
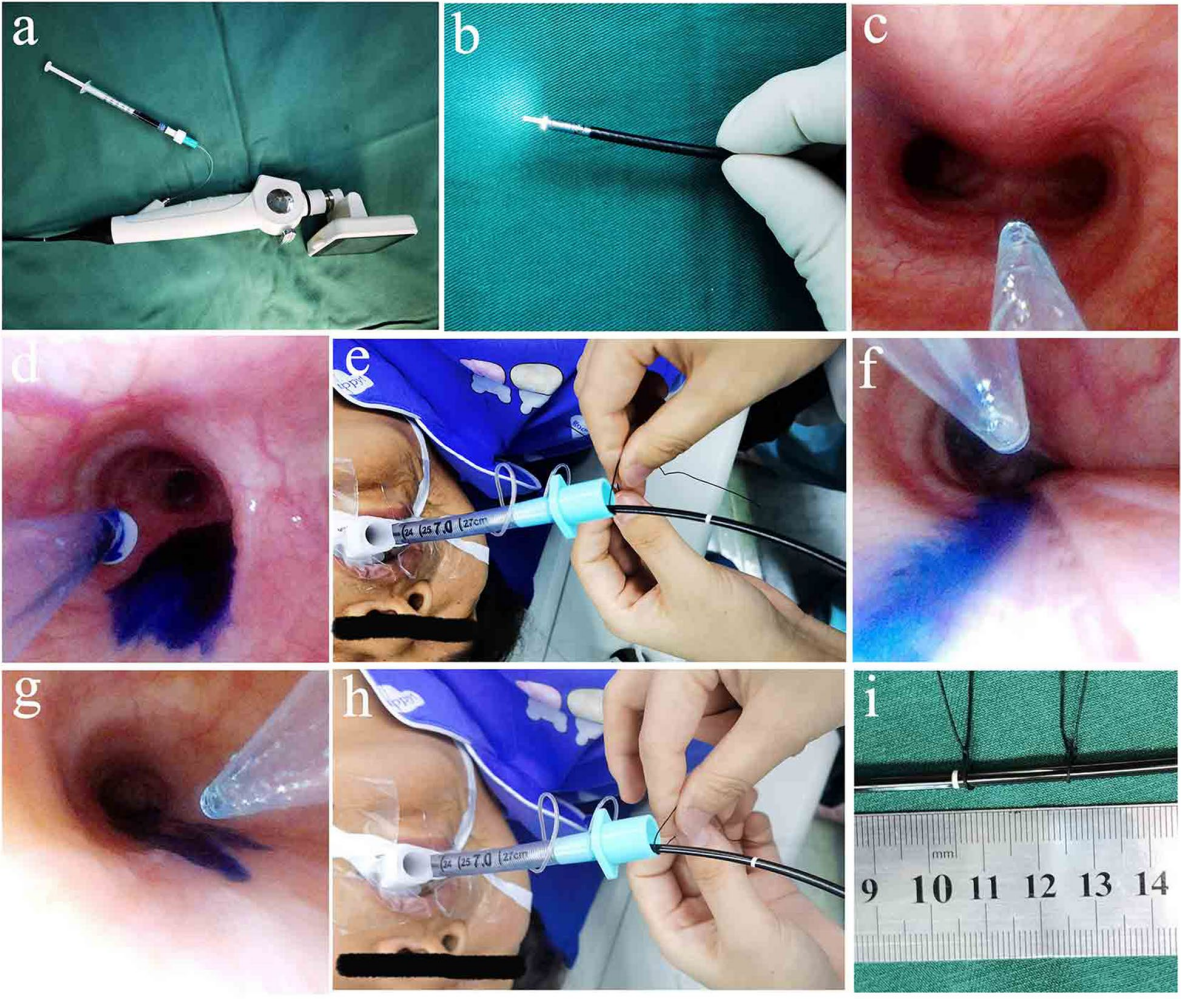
Method for measuring bronchial mucus transport velocity (BTV). **a** An epidural catheter is inserted through a biopsy hole. **b** The catheter extends from the distal end of the biopsy hole. **c** Epidural catheter reaches the junction of the left upper bronchial opening and left lower bronchial opening. **d** Inject methylene blue dye. **e** Mark the zero point. **f** Methylene blue motion trajectory. **g** The catheter moves to the near edge of the dye's trajectory. **h** Mark the mark point. **i** Measure the distance between the mark point and the zero position

The respiratory circuit was disconnected at T0, T1, and T2 time points as described by Seo H and Kim SH et al. [12, 15]. (Fig. 1). A fibrescope (Olympus BF-P40, Tokyo, Japan) was inserted through the trachea tube. An 18-gauge epidural catheter was inserted through the biopsy hole of the fiberscope, and the catheter was extended 0.5 cm from the distal end of the biopsy hole. The holes in the ends and sides of the epidural catheter were cut to ensure that the injected dye could be restricted to a single point on the surface of the airway. The fibrescope was inserted into the left main bronchus to ensure that the distal end of the epidural catheter was fixed about 0.5 cm above the junction of the left upper lobe bronchial opening and the left lower lobe bronchial opening. A 1 mL sterile syringe was used to inject a drop of methylene blue dye through the epidural catheter at 5 o ‘clock under direct vision. The blue dye was mixed with twice the volume of hydroxyethy starch solution to increase viscosity and prevent excessive dye diffusion on the airway surface that may result in a large error in BTV measurement. The injection was controlled within 1 min, and the junction between the fiberscope and the endotracheal tube opening was marked as the zero point, The fibrescope was then removed. Furthermore, it is crucial to ensure that the lens of the fiberscope does not come into contact with the injection site of methylene blue on the airway mucosa during the procedure. The position of the proximal margin of the dye was determined at 2,4,6 min after dye application, the junction between the fiberscope and the endotracheal tube opening was marked as the first, second- and third-mark points, the distance (length of the methylene blue trace) between the three marker points and the zero point was measured. The distance was divided based on the time between the phase intervals. The average value of the three results was taken as the BTV value (unit: mm/min) to reduce error.

Data were collected at the following time points:

- Baseline; 5 min after endotracheal intubation and before initiation of the pneumoperitoneum (T0)
- Post pneumoperitoneum (1 h after complete CO_2_ insufflations) (T1)
- Post pneumoperitoneum (2 h after complete CO_2_ insufflations) (T2)

BTV at T0, T1 and T2 was the primary outcome. The secondary outcomes included heart rate (HR), mean arterial pressure (MAP), body temperature, PETCO2, VT, PIP, mean inspiratory pressure (P mean), respiratory rate (RR), and dynamic compliance (CDyn) at T0, T1, and T2.

### Sample size

PASS 15 Power Analysis and Sample Size Software (2017NCSS, LLC. Kaysville, Utah, USA) was used to determine the minimum number of samples. For BTV, a minimum number of 16 patients per group was required to detect a difference of at least 5.0 mm/min with a two-tailed alpha error of 0.05 and desired power of 80% (according to the data (mean, standard deviation) of a previous investigation) [15]. Assuming a 20% dropout rate, the final sample size was increased to 20 patients per group.

### Randomization and blinding

The 66 subjects were divided into two groups (1:1) using SPSS 26.0 software program (IBM SPSS Statistics, Chicago, Illinois, USA). and computer random number method. 33 pairs of numbers (1 or 2) were randomly generated, where 1 and 2 corresponded to the PCV-VG and VCV groups, respectively. The intervention methods adopted for PCV-VG or VCV groups were stored in opaque envelopes numbered 1 to 66 in the order in which they were produced. An anesthesiologist blinded to the study opened the envelope and determined the ventilation mode (PCV-VG or VCV). Fibrescope was performed by a senior and experienced anesthesiologist blinded to the study. A nurse who was blinded to the study recorded the data. The parameters of the anesthesia machine were covered using an opaque cloth during the bronchoscopy operation to ensure the operator was blinded to the study.

### Statistical analyses

Normal distribution of data was assessed using the Shapiro-Wilktest test. The equality of group variances was evaluated using the homogeneity of variance test (Levene statistic). Two independent samples t-tests were used to analyze data for age, height, BMI, weight, anesthesia time, pneumoperitoneum time, forced expiratory volume in 1st second (FEV1), and forced vital capacity (FVC). Fisher’s exact test was used to compare the ASA physical status and cancer types. The data of HR, MAP, body temperature, V_T,_ and ETCO2 were normally distributed. Repeated measure analysis of variance was utilized to compare different variables at the study time points using the General Linear Model regression analysis with repeated measure, followed by paired t-test (as a post hoc test). The data of PIP, Pmean, RR, Cdyn, and BTV were non-normally distributed data. Wilcoxon rank-sum test was used for comparisons between the two groups for continuous variables w reported with the Wilcoxon/Mann -Whitney U test statistic. Kruskal–Wallis H test was used to compare continuous variables at different time points within the same group and reported with the Kruskal -Wallis one-way analysis of variance test statistics. Normally distributed data were presented as mean (SD) while non-parametric data were presented as the median [25%-75% percentile]. The difference in the BTV between the two groups was also assessed using box and whiskers. *P* < 0.05 was considered statistically significant. The SPSS 26.0 software program was used for all statistical analyses.

## Results

**Fig. 2:**
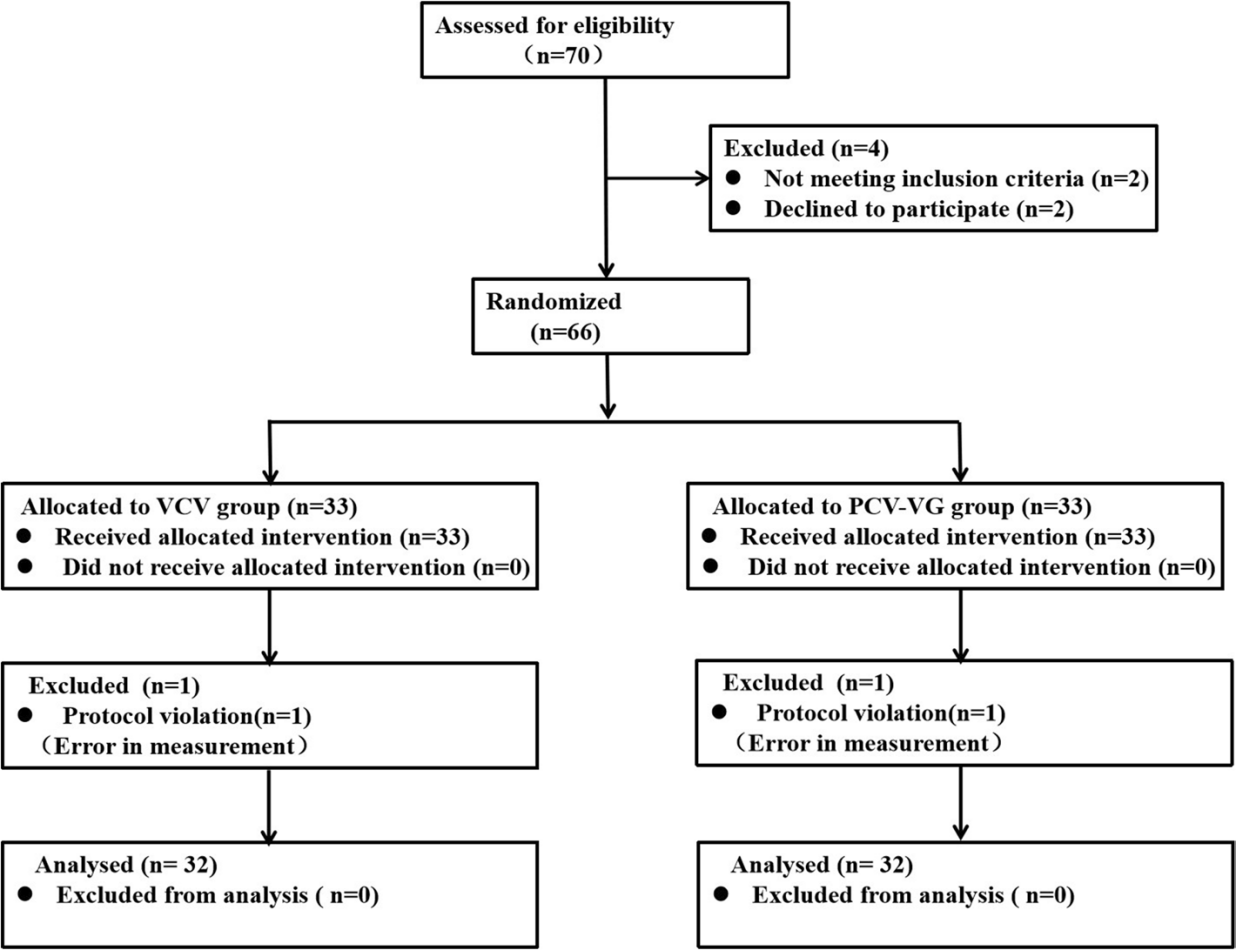
Consort diagram for patient recruitment in the trial

A total of 70 patients were recruited in the study, of which 4 were excluded (2 patients not meeting inclusion criteria and 2 declined to participate). Two of the remaining 66 patients were further excluded for protocol violation (error in measurement, one in each group). Finally, 64 participants (32 in each group) were enrolled in the study. The CONSORT diagram of the study is shown in Fig. 2. The patient characteristics, preoperative pulmonary functions, and operative data were similar between the two groups (Table 1).

**Table 1:** Patient characteristics, preoperative pulmonary functions and operative data

Baseline characteristics	VCV *N* = 16	PCV-VG *N* = 16
**Age (yr),mean (SD)**	**54.5(6.2)**	**54.3(5.5)**
**Height (cm), mean (SD)**	**160.5(2.9)**	**160.9(2.8)**
**Weight (kg), mean (SD)**	**65.9(4.3)**	**66.6(3.8)**
**BMI (kg m-2), mean (SD)**	**25.5(1.7)**	**25.7(1.7)**
**ASA physical status, median [IQR]**	**1 (1-2)**	**1 (1-2)**
**Duration of anesthesia (min),mean (SD)**	**228(46)**	**230(49)**
**Duration of pneumoperitoneum (min),mean (SD)**	**204(47)**	**206(50)**
**Preoperative FVC (% of predicted)**	**87.4(3.1)**	**86.4(2.2)**
**Preoperative FEV1 (% of predicted)**	**90.3(3.0)**	**90.3(3.0)**
**Infusion volume during the operation(ml), mean (SD)**	**2375(216)**	**2219(253)**
**Urine output during the operation(ml), mean (SD)**	**722(53)**	**698(64)**
**Cancer types, n/total (%)**		
● **Cervical cancer**	**9(56.3%)**	**8(50.0%)**
● **Endometrial cancer**	**4(25.0%)**	**5(31.3%)**
● **Ovarian cancer**	**3(18.8%)**	**3(18.8%)**

Data are presented as mean (SD), median [IQR] or n/total (%)

There were no significant differences between groups

*BMI* Body mass index, *ASA* American Society of Anesthesiologists, *FVC* Forced vital capacity, *FEV1* Forced expiratory volume in 1st second, *SD* Standard deviation, *IQR* Interquartile range

Furthermore, hemodynamic parameters and body temperature were not significantly different between the two groups (Table 2).

**Table 2:** Hemodynamic parameters and body temperature at each time point

	T0		T1		T2	
Variables	VCV group	PCV-VG group	VCV group	PCV-VG group	VCV group	PCV-VG group
	*N*=32	*N*=32	*N*=32	*N*=32	*N*=32	*N*=32
HR(beat/min),mean (SD)	66.1（6.9）	65.2（6.6）	65.4(4.9)	65.2(5.7)	66.6(5.3)	66.4(5.3)
MAP (mmHg)	81.4(7.0)	78.3(6.6)	79(7.3)	80(7.6)	80.5(7.4)	79.6(7.5)
Body temperature;℃	36.6(0.1)	36.5(0.1)	36.4(0.1)	36.4(0.1)	36.4(0.2)	36.4(0.1)

Data are presented as mean (SD) or ℃

There were no significant differences between groups

*HR* Heart rate, *MAP* Mean arterial pressure, *SD* Standard deviation

Similarly, the change in V_T_ over time did not differ between the groups. ETCO2 significantly increased in both groups during pneumoperitoneum (*P* < 0.05) (increasing with time). Also, RR increased to compensate for the increased ETCO2 (< 0.05), indicating that ETCO2 and RR had the same trend. VT was stable in the two groups throughout the procedures (*P* > 0.05). Compared with baseline (T0), PIP significantly increased at T1 and T2 in both groups (*P* < 0.05). However, PIP was significantly lower in the PCV-VG group than in the VCV group at the same time points (*P* < 0.05). Compared with T1, Cdyn significantly decreased in both groups at T1 and T2 (*P* < 0.05), but it was higher in PCV-VG group than in the VCV group (*P* < 0.05). In contrast, Pmean significantly increased in both groups at T1 and T2 compared withT0 (*P* < 0.05). However, Pmean was not significantly different between the two groups (*P* > 0.05) (Table 3).

**Table 3:** Respiratory mechanics and BTV measured at each time point

	T0		T1		T2	
Variables	VCVgroup	PCV-VGgroup	VCVgroup	PCV-VGgroup	VCVgroup	PCV-VGgroup
	*N*=32	*N*=32	*N*=32	*N*=32	*N*=32	*N*=32
VT(mL),mean (SD)	435.9（13.7）	436.2（13.4）	436.2（13.4）	430.3（16.9）	433.4（11.3）	436.2（13.4）
ETCO2(mmHg),mean(SD)	33(2.1)	32.8(2.1)	39.4(1.3)*	39.2(1.6)*	42.8(1.8)*^#^	42.5(2.6)*^#^
PIP(cmH2O),median [IQR]	13[12-14]	13 [12.3-14]	31.5 [29.3-33.8]*	23 [21.3-24]*^&^	32 [31-34.8]*	24 [23-25.8]*^&^
Pmean(cmH2O),median [IQR]	6 [5-7]	6 [6-7]	9 [8.3-9.8]*	9 [8-9]*	9.5 [9-10]*	9 [8-9]*^&^
RR(breath/min),median[IQR]	10.5 [10-11]	10 [10-11]	13.5 [13-14]*	13 [13-14]*	16 [15-17]*#	15 [14.3-16.8]*#
Cdyn(mL/cmH2O),median[IQR]	34 [30-36.8]	33 [30.5-34]	13 [12-14]*	18.5 [17.3-20]*^&^	13 [12-14]*	18 [17-19]*^&^
BTV(mm/min),median[IQR]	10.7[8.5-14.4]	10.4 [9.0-14.1]	6.8 [5.2-9.5]*	8.3 [6.8-11.3]	4.4 [3.3-5.9]*^#^	7.8[6.1-10.4]*^&^

Data are presented as mean (SD) or median [IQR]

*VT* Tidal volume, *ETCO2* End tidal CO2, *PIP* Peak inspiratory pressure, *P mean* Mean inspiratory pressure respiratory rate, *Cdyn* Dynamic lung compliance, *BTV* Bronchial mucus transport velocity, *SD* Standard deviation, *IQR* Interquartile range, *T0* Immediately after endotracheal intubation, hemodynamics and mechanical ventilation were stable, *T1* 1 h after pneumoperitoneum, *T2* 2 h after pneumoperitoneum, *P value < 0.05 compared with value of T0 in each group, # *P* value < 0.05 compared with value of T1 in each group & *P* value < 0.05 compared with the VCV group at the same time point

**Fig. 3:**
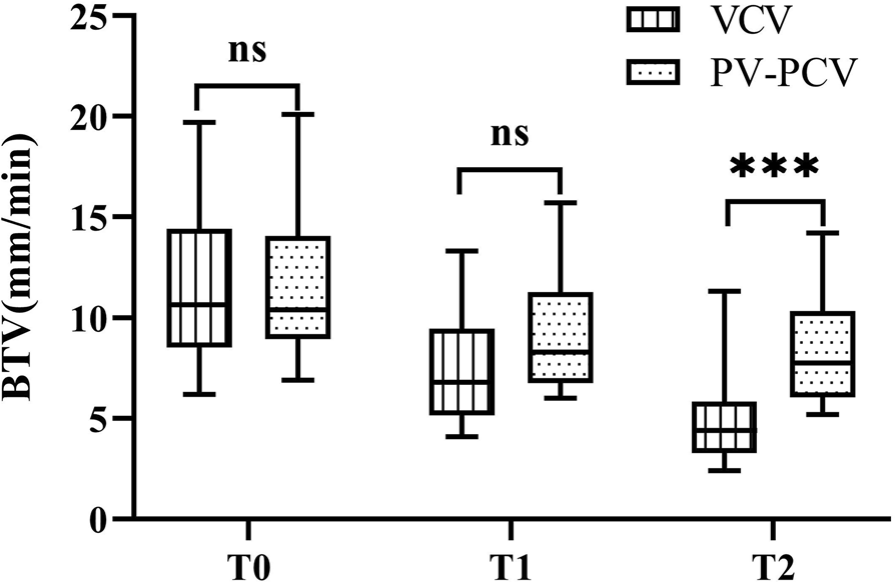
Boxplots of BTV in T0, T1 and T2 for VCV, and PCV-VG group. The solid horizontal lines indicate the medians, the boxes extend to the IQR, the whiskers extend to the maximum and minimum values of BTV T0 = Immediately after endotracheal intubation, hemodynamics and mechanical ventilation were stable; T1 = 1 h after pneumoperitoneum; T2 = 2 h after pneumoperitoneum; BTV = bronchial mucus transport velocity; IQR = interquartile range; ns: *p* > 0.05, *p < 0.05, **p < 0.01, ***p < 0.001

The median [interquartile range] of BTV was significantly lower in the VCV group at T1 and T2 than at T0 (*P* < 0.05). However, BTV was slightly lower in PCV-VG than in the VCV group. Compared with T0, BTV in the PCV-VG group was significant -ly decreased at T2 (*P* < 0.05), while BTV at T1 was slightly decreased (*P* > 0.05). Compared with the PCV-VG group, BTV in the VCV group was significantly decreased at T2 (*P* < 0.05) (Table 3 and Fig. 3). No participant experienced respiratory complications after one week of follow-up.

## Discussion

In this study, both VCV and PCV-VG ventilation modes inhibited BTV in patients during laparoscopic surgery for gynecological oncology, and the degree of inhibition increased with increasing pneumoperitoneum time. The degree of BTV inhibition was not significantly different between the two groups at 1 h after pneumoperitoneum. However, the degree of BTV inhibition was significantly less in the PCV-VG group than in VCV group at 2 h after pneumoperitoneum. In addition, VT was similar for both modes indicating that PCV-VG could deliver a preset ventilation volume to the patients. To the best of our knowledge, this is the first study to evaluate the effect of PCV-VG mode on BTV in patients during laparoscopic surgery.

Similarly, an animal study of Piccin VS comparing the effects of four mechanical ventilation strategies (normal volume ventilation, low volume ventilation, high volume ventilation, and high-pressure ventilation) on the mucociliary system [16] showed that the strategies can change distal lung, morphology, and physiology of the trachea at 3 h after mechanical ventilation, which promotes the dysfunction of the mucociliary system. Particularly, the high-pressure ventilation mode significantly damaged ciliated cells and reduced mucus secretion, thereby decreasing the BTV, consistent with this study. This effect may be ascribed to the oxidative stress induced by exposure of bronchial epithelial cells to mechanical forces generated by mechanical ventilation, which promotes the production of reactive oxygen species (ROS). ROS triggers a compression reaction of the tracheal epithelium, leading to goblet cell damage and a decrease in mucus volume [17]. In addition, the trachea is susceptible to impact forces generated by shear flow during mechanical ventilation. The high- pressure ventilation mode also increases the impact of shear forces at high flow rates, thus resulting in cell damage [18, 19]. Mechanical ventilation also decreases mucociliary transport due to dehydration, loss of cilia, and pulmonary infections [20]. In this study, PCV-VG produced significantly lower PIP and higher dynamic compliance than VCV. Other studies have also reported that the PCV-VG mode enables the ventilator to automatically compensate for changes in lung mechanism to maintain the set V_T_ during laparoscopic procedures where sudden changes in intra-abdominal pressure may occur. Moreover, changes in anesthesia depth, muscle relaxation, and surgical manipulations can alter both lung compliance and airway resistance [21]. The VCV mode cannot adapt to this change, while the PCV-VG mode can effectively combine the advantages of volume-control and pressure-limited ventilation [21, 22]. In the PCV-VG mode, V_T_ and RR are preset, and the first ventilation of the anesthesia machine is based on the preset VT. The patient’s respiratory compliance is dependent on the last breath volume, whereas the pressure level for the next breath is influenced by the calculated compliance. In summary, PCV-VG ventilation provides the minimum airway pressure to achieve the desired VT based on the actual respiratory mechanics [23]. This explains the slight decrease in BTV after pneumoperitoneum via PCV-VG mode.

Jung Min Lee et al. [21]. compared VCV, PCV, and PCV-VG modes during robot -assisted laparoscopic gynecologic surgery in the Trendelenburg position and reported that the PCV-VG mode can slightly change airway pressure compared with the VCV mode, and thus could reduce barotrauma and airway damage. They also indicated that the flow velocity of PCV-VG exhibits a decelerating flow pattern. The flow velocity is fast at first, then slows, and decreases exponentially, while the preset pressure is maintained throughout the inspiratory interval. The airway pressure wave is square, and the gas distribution is more uniform. However, VCV is a constant airflow velocity, and airway resistance changes greatly during mechanical ventilation, and thus may significantly inhibit BTV after pneumoperitonea [24].

Although mechanical ventilation is widely used in intubated patients with various medical and surgical conditions, its direct effect on BTV in patients undergoing anesthesia is unknown. In this study, fiberscope was used to locate, map, and measure BTV. This method compares with the radioactive aerosol inhalation lung scanning method, nasal saccharin transport time test, and other methods for determining and evaluating BTV. Besides, it is a relatively accurate and non-invasive measurement method [25]. Measurements can be taken using a fiberoptic bronchoscope, even down to the level of the subsegmental bronchus, at ease without much concern about hypoxia since the patient is supported by an anesthesia machine during the procedure. Furthermore, an execution team ensured that the fibrescope operator only measured BTV, the anesthesiologist was only responsible for setting the ventilation mode, and the nurse was only responsible for data collection.

However, this study has some limitations. First, the movement trajectory of methylene blue dye on the airway surface of most patients under general anesthesia was not an absolute straight line but showed an irregular curve. This indicates that although the total swing direction of the airway surface cilia is toward the direction of throat, each swing does not always go in a straight line [26]. In addition, the airway surface mucosa has a viscous resistance effect; thus, airway secretions are subjected to various forces during the movement process. These factors explain the curved movement trajectory of methylene blue stain on the airway surface. Second, the enrolled patients were American Society of Anesthesiologists physical status 1 and 2, low-risk patients with normal pulmonary function before surgery, with no history of respiratory disease, and pneumoperitoneum duration was only 2 h. Thus, these results are only applicable to patients without respiratory complications. Nevertheless, further studies should assess whether patients with basic respiratory diseases or long-term pneumoperitoneum may develop respiratory complications after surgery. Third, the BTV values of patients were not collected before endotracheal intubation. Thus, BTV values measured 5 min after endotracheal intubation and before pneumoperitoneum were used as a baseline. The recorded BTV value was sufficiently high, indicating that mucociliary clearance remained unaffected during endotracheal intubation. Fourth, although the method for BTV measurement is simpler and more intuitive than the previous measurement methods, it requires skilled doctors during the operation process. Improper handling of the fibrescope lens may damage the airway mucosa or cause measurement errors. Fifth, this is an in-vivo analysis, which is not as accurate as the in vitro research since the ultrastructure of the cilia cannot be observed, and the cilia swing frequency cannot be measured for accurate assessment of the ciliary movement status, and analysis of the overall condition of the ciliary movement. Only a dynamic process was observed due to res cilia transporting mucus from in-vivo to in-vitro through the bronchus (the actual movement length of methylene blue trace).

## Conclusions

Although both PCV-VG and VCV inhibit BTV after 2 hours of pneumoperitoneum, the BTV was significantly higher in the PCV-VG group than in the VCV group. Therefore, PCV-VG mode can effectively protect mucociliary clearance function in patients undergoing laparoscopic surgery for gynecologicaloncology. This is likely due to the fact that PCV-VG enables ventilation at lower PIP and offers greater dynamic compliance compared to VCV, resulting in reduced potential for damage to the airway mucosa.

## Data Availability

The datasets used and analyzed during the current study are available from the corresponding author on request.
